# Molecular characterisation and epidemiology of transmission of intraoperative *Staphylococcus aureus* isolates stratified by vancomycin minimum inhibitory concentration (MIC)

**DOI:** 10.1016/j.infpip.2022.100249

**Published:** 2022-09-08

**Authors:** Brent Hadder, Franklin Dexter, Alysha D.M. Robinson, Randy W. Loftus

**Affiliations:** Department of Anaesthesia, University of Iowa, Iowa City, IA, USA

**Keywords:** MRSA, Vancomycin, VISA, Transmission, Epidemiology, Intraoperative

## Abstract

**Background:**

Reduced vancomycin susceptibility in *Staphylococcus aureus* (*S. aureus*) is considered a more pathogenic strain characteristic and is associated with treatment failure. We aimed to characterise the epidemiology of intraoperative transmission of *S. aureus* isolates with reduced vancomycin susceptibility.

**Methods:**

*S. aureus* isolates (N=173) collected from 274 randomly selected operating room environments at three major academic medical centres in 2009–2010 were characterised by vancomycin minimum inhibitory concentration (MIC). We aimed to characterise the transmission dynamics for VISA and isolates with relatively reduced vancomycin (MIC= 2μg/mL) susceptibility at the range of therapeutic differentiation.

**Results:**

Intraoperative *S. aureus* MIC was 1.38 ± 0.34 μg/mL. No VISA isolates were identified (95% upper confidence limit 2.1%) and those with an MIC of 2 μg/mL accounted for 12.72% (22/173) of all isolates. MIC=2 μg/mL isolates were more frequently cultured from the hands of healthcare providers [19.3% (16/83)] versus otherwise [6.7% (6/90)], with unadjusted risk ratio 2.89, *P*=0.021, and from patients with >2 major comorbidities [25.0% (8/32)] versus otherwise [9.9% (14/141)], with unadjusted risk ratio 2.52, P=0.035. Both were significant when tested simultaneously. The adjusted relative risk for provider hands was 2.77 (95% CI 1.15 to 6.69, P=0.024). The adjusted relative risk for patients with >2 major comorbidities was 2.37 (95% CI 1.11 to 5.05, P=0.026). MIC=2μg/mL was not associated with greater risk of clonal transmission (unadjusted P=0.34, adjusted P=0.18).

**Conclusion:**

Intraoperative VISA is a rare event. S. aureus isolates MIC=2μg/mL isolates were not associated with increased risk of intraoperative transmission. The epidemiology of detected intraoperative transmission is consistent with Centers for Disease Control guidelines.

## Introduction

The pathogenicity of *S. aureus* is related to ongoing acquisition of genetic traits that enhance antimicrobial resistance, virulence, and survival. *S. aureus* causes more deaths in the United States than the human immunodeficiency virus, with an attributable mortality rate estimated at approximately 6/100,000 individuals. It affects healthy members of our communities and is a leading cause for surgical site, bloodstream, and respiratory infections [[1], [2], [3], [4], [5], [6], [7], [8], [9]].

Vancomycin-intermediate *S. aureus* (VISA) and heterogeneous VISA (hVISA) with minimum inhibitory concentrations (MICs) ranging from 1-4μg/mL are associated with antibiotic treatment failure [10]. In one study, 86% of patients infected with hVISA experienced treatment failure compared to 20% of infected patients that were hVISA negative [11]. In response, the vancomycin minimum inhibitory concentration (MIC) breakpoint was reduced from ≤4 μg/mL to ≤2 μg/mL for susceptible isolates and from ≥32 μg/mL to ≥16 μg/mL for resistant strains in 2006 [12]. In a follow-up study in 2011, MIC ≥ 1.5 μg/mL was associated with slower treatment response and increased risk of treatment relapse for patients diagnosed with *meticillin-resistant S. aureus* pneumonia [13]. Thus, prevention of transmission of *S. aureus* isolates with MIC > 1.5μg/mL is an important safety consideration for all healthcare settings.

The epidemiology of intraoperative VISA transmission has not been assessed. In this study, our primary aim was to characterise the baseline epidemiology of intraoperative VISA and minimum inhibitory concentration >2 μg/mL (MIC=2) transmission at the threshold of therapeutic differentiation [10], within the reported range of heterogeneous VISA (hVISA) [10], and at a level associated with slow treatment response and increased risk of relapse [13]. We sought to evaluate the incidence of transmission of VISA and MIC=2μg/mL isolates and to identify for each of these strains characteristics including common reservoir(s) of origin, transmission locations, modes of transmission (within or between-case), and portals of entry.

## Methods

### Background

Two hundred seventy-four operating room environments were randomly selected for observation at 3 United States academic medical centres [14]. The observational unit was a case pair including the first and second case of the day in each operating room so within and between-case *S. aureus* transmission could be detected. As study activity was limited to analysis of anonymised data from the previous IRB approved project (201507774, Assessment of Routine Intraoperative Horizontal Transmission of Potentially Pathogenic Bacterial Organisms II), the University of Iowa declared that the additional analysis in the current study did not meet the definition of human subject's research.

Infection control practices included routine and terminal environmental cleaning with quaternary ammonium compounds ± surface disinfection wipes. All providers had access to alcohol dispensers for hand hygiene located on the wall and/or anaesthesia carts, and gloves were immediately available for use. There were no changes in these usual procedures during the study period [14].

### Overview of *S. aureus* reservoir collection process among study units

*S. aureus* isolates (N=173) were recovered from operating room reservoirs including environmental sites (baseline and at case end), healthcare provider hands throughout care, and patients after induction of anaesthesia and stabilisation. Patients were followed for 30 days to assess for development of healthcare-associated infections (HCAI). *S. aureus* isolates identified as causative organisms of infection were compared to intraoperative isolates obtained during the time of surgery.

### Isolate analysis (N=173)ETEST® glycopeptide-resistance detection by MIC (bioMérieux, marcy l’Etoile, France)

Pure isolates were streaked on blood agar plates (Remel, Lenexa, KS) and incubated 18–24 hours at 37°C. The 18–24-hour colonies were used to make 0.5 McFarland dilutions in 3 mL tubes of sterile normal saline. Sterile cotton swabs were used to dip into the suspension and were turned along the side of the tube to remove excess liquid from the swab. Swabs were then used to cover the entire surface of 100 mm Mueller Hinton agar plates (Remel, Lenexa, KS) using a zigzag technique three times, turning the plate 60 degrees each time to ensure total plate coverage with the inoculum. The plates were allowed to dry for approximately 15 minutes. The vancomycin ETEST® strips were then applied to the centre of the plate using sterile forceps, and the plates were incubated 20–24 hours at 35°C. The MIC values were read at the pointed end of the area of inhibition and recorded [15,16].

### Antibiotic susceptibility

Disk diffusion antibiotic susceptibility testing (meticillin, ampicillin, cefazolin, cefepime, ceftazidime, cefuroxime, ciprofloxacin, clindamycin, gentamicin, meropenem, penicillin, piperacillin-tazobactam, sulfamethoxazole-trimethoprim, linezolid, tetracycline, and vancomycin) analysis was done for commonly employed prophylactic antibiotics. Meticillin-resistant *S. aureus* (MRSA) was confirmed by agar dilution minimum inhibitory concentration (Remel Spectra ™ MRSA or Remel ™ Mannitol Salt Agar w/Oxacillin (4μg/mL), Lenexa, KS 66215). Antibiotic susceptibility results were used along with results of analytical profile indexing and temporal association to identify epidemiologically related isolates (same class of pathogen in two distinct reservoirs with the same analytical profile index number and same antibiotic susceptibility pattern for each of the above antibiotics)where 0=sensitive, 1=intermediate, and 2=resistant. Epidemiologically related isolates were further compared with whole cell genome analysis as described below to identify clonally related isolates [9,14,17].

### Bacterial identification

The microbiological methods used for bacterial identification included Gram stain, simple rapid tests, the commercially available bioMerieux API ® identification system (Marcy l’Etoile, France), and whole genome analysis [9,14,17].

### Multilocus sequence testing

DNA was extracted, and next generation sequencing was performed at the Iowa Institute of Human Genetics (IIHG) using the Illumina platform. DNA samples (1.2ug/60ul) were sheared to ∼400bp fragments on the Covaris E220. Sequencing libraries were prepared from the sheared DNA (1ug/50ul) using the KAPA Hyper Library Prep on the PE Caliper Sciclone (Rosche Diagnostics, Indianapolis, IN 46250–0457). Each library was prepared using an adapter that carries a unique barcode (Integrated DNA Technologies, Coralville, Iowa 52241). Libraries were analysed on a fragment analyser and equimolar amounts of the libraries were pooled based on fragment analyser results for a smear analysis of 450–670bp. A size range of 450–670bp was recovered from the pool on the Blue Pippin (Sage Science, Beverly, MA 01915). The KAPA library quantification kit for Illumina platforms was used to determine the molar concentration of the size-selected pool. The pool was loaded on the cBot (Illumina) for cluster generation and the flow cell loaded on the HiSeq4000 (Illumina) for sequence analysis [18,19].

*S. aureus* sequences reads were generated and downloaded into the CLC Genomics Workbench Module (Version 1.1, Qiagen Aarhus, Germantown, MD 20874). CLC Genomics Workbench Plugin (CLC Genomics Module Version 1.1, Qiagen Aarhus, Germantown, MD 20874) was used to trim and to remove adapters and broken pairs from *S. aureus* sequence reads, and K-mer spectra analysis was utilized to identify a best match to *S. aureus* isolates. *S. aureus* 252 (MRSA252, NC_002952) was identified as the best reference sequence match. All trimmed *S. aureus* sequence reads were subsequently mapped to the MRSA252 complete genome. Consensus sequences for each read map were analysed by multilocus sequence typing analysis (CLC Genomics Module Version 1.1, Qiagen Aarhus, Germantown, MD 20874) [18,19]. Antimicrobial resistance genes identified with the microbial genetics module. Resistance genes assessed included *spc, aadD*, *aph3III*, *aac6aph2*, *ant6Ia*, *mecA, blaZ*, *ermA, ermC, mphC, InuA*, *msrA*, *tetM, tetK,* and *norA* [[20], [21], [22]].

### Transmission

*S. aureus* isolates present at case end that were not present at case start were considered transmitted. Clonality was only considered if there were ≥ 2 isolates. Temporal association, analytical profile indexing, antibiotic susceptibility testing, multilocus sequence typing, and single nucleotide variant analysis were used to compare ≥ 2 isolates obtained from distinct reservoirs within an observational unit to characterise transmission dynamics for transmitted pathogens. Greater than 99.99% agreement in single nucleotide variants (SNV) was required for clonal relatedness, which corresponded to 49±26 SNV differences isolates [23].

Clonally related VISA and MIC=2μg/mL isolates were characterised to identify reservoirs of origin, transmission locations, modes of transmission, and portals of entry.

### Sample collection technique

#### Hand sampling

A modified glove juice technique was utilised to sample provider hands before, during, and after patient care [14].

#### Patient sampling

The patient's nasopharynx was sampled to assess the patient reservoir because nasopharyngeal pathogens have been strongly associated with postoperative surgical-site infections. The patient's axilla was also sampled because the axilla harbours up to 15%–30% of pathogens colonising patient skin [14].

#### Environmental sampling

The adjustable pressure-limiting valve and agent dial of the anaesthesia machine were sampled. These sites have been previously associated with an increase in the probability of bacterial contamination of patient intravenous stopcock sets [14]. Sites were sampled at baseline after active decontamination at case start for case 1 and after routine decontamination at case start for case 2. They were sampled again at the end of the case 1 and case 2 via Dimension III (Butcher's, Sturtevant, WI) disinfectant solution according to manufacturer's recommendations. Active decontamination involved targeted cleaning of the study sites by the study investigators using a quaternary ammonium compound strictly according to the manufacturer's protocol allowing 10 minutes for air drying which was not mandated for routine cleaning [14].

### Microbial culture conditions

All cultures were performed in the same laboratory at Site 0. Samples shipped from Sites 1 and 2 were placed under similar environmental conditions (ambient temperature) during the 12 hours required for shipping. Samples collected on the same day at Site 0 did not require shipping but were kept at ambient temperature to mimic the environment of those samples being shipped. No samples for a given study day were incubated until all samples for that day from all research sites were present at Site 0 [14].

### Postoperative infections

All patients were for followed for 30 days in the postoperative period for infection surveillance [14]^.^ Initial screening included elevated white blood cell count, fever, anti-infective order, culture, or office documentation of signs of infection. If ≥ 1 one criteria were present, the patient underwent a full chart review by the principal investigator at each hospital site to determine whether patients met criteria for a healthcare-associated infection (HCAI) as defined by the National Healthcare Safety Network [24]. All cultures obtained for infection workup were saved for comparison to intraoperative reservoir isolates with systematic phenotypic, pulsed-field gel electrophoresis, and single nucleotide variation analysis.

### Demographic data

Basic patient, procedural, and provider demographic information collected including the hospital [labelled 0, 1, or 2], age > 50 years old, sex, American Society of Anaesthesiologists physical status >2, patients with >2 comorbidities, type of surgery (cardiothoracic/vascular, orthopaedic, gynaecology/oncology, general abdominal, plastics/breast, and other), dirty or infected site, duration of greater than 2 hours, Study on the Efficacy of Nosocomial Infection control (SENIC) score (an index predicting the probability of postoperative HCAI development for a given patient) [25] >2, urgent surgery, and patient origin and discharge locations including the hospital floor, intensive care unit, or post anaesthesia care unit**Statistical Analysis**.

*S. aureus* isolate MIC values for all 173 isolates and the incidence of MRSA were summarised according to simple descriptive statistics. The two-sided confidence interval for the incidence of intraoperative VISA was calculated using the Clopper-Pearson method.

Clonally related transmission events involving VISA and MIC=2μg/mL isolates were summarised. Fisher's exact test was planned to be used to compare the frequency of reservoir (provider hands, patient skin sites, and environmental surfaces) isolation of VISA and MIC=2 as compared to all other isolates. There was however isolation of only MIC=2 isolates.

Adjustment for potentially confounding variables was planned. To do so, Fisher-exact tests were used to examine the potential association of the above demographic variables with MIC isolates=2, except age, which was tested using a t-test. The one covariate with P<0.15 was used for adjustment, specifically patients with >2 comorbidities. Poisson regression with robust variance was then used to estimate the incidence risk ratio (IRR) of MIC isolates=2μg/mL for the independent variables (i) hand isolation or not and (ii) patients with greater than 2 comorbidities or not. Poisson regression was used to estimate the risk ratio because the incidence of transmission (33.512.7%, N = 5822/173) was so large that the odds ratio estimated using logistic regression would be a biased estimator of the relative risk [[26], [27], [28]].

The relative risk of transmission for isolates with a MIC=2μg/mL as compared to all other intraoperative isolates was assessed. The association between any clonal transmission event and each of the above listed demographic covariates was checked. Poisson regression with robust variance was then used to estimate the incidence risk ratio (IRR) of any transmission event for the independent variable of MIC=2μg/mL or not, while adjusting for the covariates with P < 0.15, specifically American Society of Anaesthesiologists physical status > 2, general abdominal surgery, site 2, and duration of > 2 hours.

Antibiotic resistance, resistance traits, and MLST (1, 5, 8, 20, 30, 50, 59, 15, 72, 105, 188, and 1049) for isolates with a MIC=2μg/mL were compared to all other intraoperative isolates and assessed via the Fisher's exact test. With 15 antibiotics and 15 resistance traits, P<0.0033 was considered statistically significant, where 0.0033 = 0.05/15.

All *S. aureus* isolates (N=173) collected within demographic units were included in this study. The statistical analyses were performed using STATA 17.1. All P-values and confidence intervals are two-sided.

## Results

Ninety-nine case pairs were observed at site 0, 72 at site 1, and 103 at site 2. Sixty-four, 37, and 72 *S. aureus* isolates were collected from the case pairs at each respective site.

The overall MIC average for intraoperative *S. aureus* isolates was 1.38 ± 0.34μg/mL, with a range of 1.0–2.0 μg/mL. Zero VISA isolates were detected. Isolates with a MIC of 2μg/mL accounted for 12.72% (22/173) of all isolates. The overall incidence of case-pair MRSA exposure was 9% (25/274 operating rooms), specifically 11% (11/99) at site 0, 7% (7/72) at site 1, and 9% (9/103) at site 2. A total of 24 isolates had MIC ≥ 2, and 18% (4/22) were MRSA. A total of 109 isolates had MIC ≥ 1.5, and 27% (29/109) were MRSA.

MIC=2μg/mL was not associated with greater risk of clonal transmission. The unadjusted RR = 0.65, 95% CI = 0.29 to 1.44, P = 0.34. Adjusting for potential covariates, RR = 0.58, 95% CI = 0.27–1.27, *P*=0.18) (Table I)

**Table I:** Assessment of the potential association of MIC=2μg/mL S. aureus isolates and genomic transmission

Transmission genomic	Risk ratio	P-value	95% CI
MIC=2	0.58	0.18	0.27–1.27
ASA > 2	1.47	0.06	0.98–2.21
General abdominal surgery	0.50	0.05	0.25–0.99
Hospital site two	0.78	0.29	0.49–1.23

MIC=2: vancomycin minimum inhibitory concentration 2 μg/mL.

ASA: American Society of Anesthesiologists' physical status.

CI: Confidence interval.

The epidemiology of transmission of *S. aureus* MIC=2μg/mL isolates in summarised in Table II. MIC=2μg/mL isolates were more frequently cultured from provider hands [19.3% (16/83)] versus otherwise [6.7% (6/90)], with unadjusted risk ratio 2.89, P=0.021. They also were more frequently cultured from patients with > 2 major comorbidities [25.0% (8/32)] versus otherwise [9.9% (14/141)], with unadjusted risk ratio 2.52, P=0.035. Testing both covariates simultaneously, the adjusted relative risk for provider hands was 2.77 (95% CI 1.15 to 6.69, P=0.024). The adjusted relative risk for patients with > 2 major comorbidities was 2.37 (95% CI 1.11 to 5.05, P=0.026). The incidence of transmission events occurring within and between cases was similar. Intravascular device contamination was confirmed.

**Table II:** Epidemiology of anaesthesia work area transmission of *S. aureus* isolates with vancomycin MIC=2μg/mL isolates (N=22 isolates, 10 Transmission events)

**Reservoir origin**^a^	
Patient N (%)	5 (22.7)
Hand N (%)	16 (72.7)^c^
Environment	1 (4.5)
**Mode for Confirmed Source**^b^	
Within-case N (%)	3 (30)
Between-case N (%)	1 (10)
**Within-case transmission Location**^b^	
Patient N (%)	0 (0)
Hand N (%)	5 (50)
Environment N (%)	0 (0)
**Between-Case Transmission Location**^b^	
Patient N (%)	2 (40)
Hand N (%)	0 (0)
Environment N (%)	0 (0)
**Intravascular Device Contamination N (%)**^b^	1 (10)

Within-case is within an operating room.

Between-case is between operating rooms.

a % is reservoir isolates/number of MIC=2μg/mL (N=22).

b % is event divided by transmitted isolates (N=10).

c MIC=2 isolates were more frequently cultured from provider hands [19.3% (16/83)] versus otherwise [6.7% (6/90)], with unadjusted risk ratio 2.89, P=0.021.

MIC=2μg/mL isolates were not significantly associated with antibiotic resistance (all P ≥ 0.024), acquisition of resistance traits (all P ≥0.024), or MLST (all P ≥0.1).

## Discussion

*S. aureus* transmission is an important target for postoperative infection prevention [[1], [2], [3], [4], [5]]. Reduced vancomycin susceptibility is a more pathogenic strain characteristic that may be an important consideration for intraoperative infection control [10]. In this study, we sought to characterise the baseline epidemiology of intraoperative *S. aureus* isolates with reduced vancomycin susceptibility. We found that intraoperative VISA was undetectable and that isolates with MIC=2μg/mL were not associated with increased risk of clonal transmission, resistance, acquisition of resistance traits or MLST.

Prior work has suggested that the overall VISA prevalence among clinical isolates in the United States is approximately 2.75% [29]. The prior work also identified an increasing trend in VISA detection among MRSA isolates, 2.05% before 2006 versus 7.01% in 2010–2014 [29]. We detected a MRSA rate consistent with healthcare worker colonisation rates of 7.5% [30], and our rate of detection for MRSA isolates with higher MIC (≥1.5μg/mL) was consistent with the literature [13]. Given these consistencies and the conceptual framework that VISA is derived from MRSA isolates [10], we expected a similar frequency of intraoperative VISA detection ranging from 2.75 to 7%. However, we were unable to find a single intraoperative isolate with a MIC >2μg/mL across three major academic medical centres; the upper 95% confidence limit was an incidence of 2.1%. These results suggest that the intraoperative VISA prevalence during our study, 0%, was lower than previously reported rates (2.75%, 95% CI 1.19–4.91) [29]. However, given that prior evidence suggests an upward trend of VISA isolation, [29] future work should re-examine intraoperative VISA rates and compare to the baseline reported in this study.

The maximal MIC we detected was 2μg/mL, which accounted for approximately 12% of isolates. Because this MIC is at the threshold of therapeutic differentiation (>2μg/mL) [10], within in the reported range of heterogeneous VISA (hVISA) [10,12], and associated with slow treatment response and increased risk of relapse [13], we explored the epidemiology of *S. aureus* MIC=2μg/mL transmission. Using whole cell genome analysis, we were able to directly link these isolates within and between-case transmission events that ultimately led to patient infection. We confirmed within and between-case modes of transmission and intravascular device contamination, a patient portal of entry. Provider hands and patient skin sites were confirmed sources of infection and patient-to-patient transmission, respectively, which are two key preventative targets for the Centers for Disease Control (CDC) [31,32].

The CDC recommends hand hygiene as the number one preventative measure for control of VISA outbreaks [31,32]. The results of our study support this statement with MIC=2 μg/mL isolates more likely to be cultured from provider hands than other intraoperative isolates. In addition, our study of transmission dynamics linked patient skin surfaces, especially those patients with >2 comorbidities, to intraoperative patient-to-patient transmission, a key target for the CDC [31,32], suggesting that improved patient decolonisation would be helpful in addition to hand hygiene in the OR for controlling this strain characteristic. Thus, in addition to optimal hand hygiene compliance, targeted patient decolonisation involving patients with two or more comorbidities may represent a best practice for perioperative control of VISA spread.

As VISA is thought to be derived from MRSA, a highly transmissible intraoperative strain characteristic [33], we hypothesised that *S. aureus* strains with reduced vancomycin susceptibility might also be highly transmissible. We were unable to support this hypothesis. Importantly, there was no association of MIC=2μg/mL with ST-239 or ST-5 as reported previously [29]. This finding is consistent with the lack of association with MRSA or resistance traits, further supporting a low risk of reduced vancomycin susceptibility. There is both a low incidence of higher vancomycin MIC and no detectable association with high-risk *S. aureus* genotypes.

Our assessments were limited by the isolates obtained from operating rooms in this study. While isolates were obtained in 2009–2010, this established baseline is important for future characterization of intraoperative VISA and MIC=2μg/mL. This is critical given confirmation of evolving worldwide resistance associated with increased patient mortality [34].

In conclusion, intraoperative VISA went undetected in this study. Our study of intraoperative transmission dynamics for isolates with a MIC=2μg/mL corroborate with CDC guidelines pertaining to VISA control.

## Credit author statement

Randy Loftus helped with conceptualization, study execution, genomic analysis, data analysis and manuscript preparation and provided oversight for microbiological processing and approved the final manuscript. Alysha Robinson conducted microbiological data processing, helped with manuscript preparation, and approved the final manuscript. Brent Hadder helped with conceptualization, manuscript preparation and approved the final manuscript. Frank Dexter helped with conceptualization, manuscript preparation, data analysis, and approved the final manuscript.
