# Supplementary material for: Molecular characterisation and epidemiology of transmission of intraoperative Staphylococcus aureus isolates stratified by vancomycin minimum inhibitory concentration (MIC)
Source: Infect Prev Pract. 2022 Sep 8;4(4):100249. doi: 10.1016/j.infpip.2022.100249 (PMC9523349; doi:10.1016/j.infpip.2022.100249)
Supplement: Multimedia component 1 [file mmc1.docx]

**Supplementary Material**

|  | **Sensitive** | **Intermediate Resistance** | **Resistant** |
| --- | --- | --- | --- |
| **Antibiotic** |  |  |  |
| **Methicillin N (%)** | 129 (74.6) |  | 44 (25.4) |
| **Ampicillin N (%)** | 24 (13.9) |  | 149 (86.1) |
| **Cefazolin N (%)** | 143 (82.7) | 2 (1.2) | 28 (16.2) |
| **Cefepime N (%)** | 140 (80.9) | 4 (2.3) | 29 (16.8) |
| **Ceftazidime N (%)** | 17 (9.8) | 73 (42.2) | 83 (50) |
| **Cefuroxime N (%)** | 141 (81.5) | 3 (1.7) | 29 (16.8) |
| **Meropenem N (%)** | 145 (83.8) |  | 28 (16.2) |
| **Penicillin N (%)** | 27 (15.6) |  | 146(84.4) |
| **Piperacillin-Tazobactam N (%)** | 114 (65.9) |  | 59 (34.1) |
| **Ciprofloxacin N (%)** | 142 (64.7) | 30 (17.3) | 31 (17.9) |
| **Clindamycin N (%)** | 84 (48.6) | 76 (43.9) | 13 (7.5) |
| **Gentamicin N (%)** | 167 (96.5) | 2 (1.2) | 4 (2.3) |
| **Trimethoprim/Sulfamethoxazole N (%)** | 171 (98.8) |  | 2 (1.2) |
| **Linezolid N (%)** | 168 (97.1) |  | 5 (2.9) |
| **Tetracycline N (%)** | 166 (96) | 2 (1.2) | 5 (2.9) |

**Table I: Antibiotic susceptibility profiles for intraoperative anaesthesia work area reservoir *Staphylococcus aureus* isolates**
